# MR Fingerprinting with b‐Tensor Encoding for Simultaneous Quantification of Relaxation and Diffusion in a Single Scan

**DOI:** 10.1002/mrm.29352

**Published:** 2022-06-17

**Authors:** Maryam Afzali, Lars Mueller, Ken Sakaie, Siyuan Hu, Yong Chen, Filip Szczepankiewicz, Mark A. Griswold, Derek K. Jones, Dan Ma

**Affiliations:** ^1^ Leeds Institute of Cardiovascular and Metabolic Medicine University of Leeds Leeds UK; ^2^ Cardiff University Brain Research Imaging Centre (CUBRIC), School of Psychology, Cardiff University Cardiff UK; ^3^ Imaging Institute, Cleveland Clinic Cleveland Ohio USA; ^4^ Biomedical Engineering Case Western Reserve University Cleveland Ohio USA; ^5^ Radiology Case Western Reserve University Cleveland Ohio USA; ^6^ Clinical Sciences Lund Lund University Lund Sweden

**Keywords:** b‐tensor encoding, diffusion imaging, magnetic resonance fingerprinting, multidimensional MRF, quantitative MR, relaxometry

## Abstract

**Purpose:**

Although both relaxation and diffusion imaging are sensitive to tissue microstructure, studies have reported limited sensitivity and robustness of using relaxation or conventional diffusion alone to characterize tissue microstructure. Recently, it has been shown that tensor‐valued diffusion encoding and joint relaxation‐diffusion quantification enable more reliable quantification of compartment‐specific microstructural properties. However, scan times to acquire such data can be prohibitive. Here, we aim to simultaneously quantify relaxation and diffusion using MR fingerprinting (MRF) and b‐tensor encoding in a clinically feasible time.

**Methods:**

We developed multidimensional MRF scans (mdMRF) with linear and spherical b‐tensor encoding (LTE and STE) to simultaneously quantify T1, T2, and ADC maps from a single scan. The image quality, accuracy, and scan efficiency were compared between the mdMRF using LTE and STE. Moreover, we investigated the robustness of different sequence designs to signal errors and their impact on the maps.

**Results:**

T1 and T2 maps derived from the mdMRF scans have consistently high image quality, while ADC maps are sensitive to different sequence designs. Notably, the fast imaging steady state precession (FISP)‐based mdMRF scan with peripheral pulse gating provides the best ADC maps that are free of image distortion and shading artifacts.

**Conclusion:**

We demonstrated the feasibility of quantifying T1, T2, and ADC maps simultaneously from a single mdMRF scan in around 24 s/slice. The map quality and quantitative values are consistent with the reference scans.

## Abstract 2

Click here for author‐reader discussions

## 1 INTRODUCTION

At typical MRI resolution, each voxel contains multiple microenvironments with different tissue properties. This challenges detection and diagnosis of many diseases that are characterized by cellular‐level heterogeneity, such as epilepsy and brain tumors. Therefore, there is an increasing interest in characterizing tissues and lesions with multiple image contrasts to improve the sensitivity to tissue microstructure. Two main categories in quantitative MRI for multi‐parametric imaging and microstructure imaging are MR relaxometry, such as T1, T2, and T2* mapping,^1^ and diffusion MRI.^2^, ^3^

To improve the sensitivity and specificity of characterizing complex tissues/lesions and translate the techniques into clinical practice, there are three main challenges that need to be addressed. First, although both relaxation and diffusion are sensitive to the microenvironment and have been commonly used in disease characterization,^4^, ^5^, ^6^, ^7^, ^8^, ^9^, ^10^, ^11^ relaxation and diffusion MRI data are typically acquired separately and analyzed using separate models, which could lead to registration errors and estimation bias. Specifically, for multi‐compartment estimation when multiple tissues with different relaxation rates and diffusion times are present, multidimensional encoding and signal models that account for relaxation‐diffusion correlations would allow decoupling of each compartment that cannot be achieved with relaxation or diffusion only methods. Studies showed that using relaxation only made the estimation of multi‐compartment models sensitive to noise,^12^ and using diffusion only to disentangle intra‐ and extra‐axonal compartment properties in brain white matter is degenerate and ill‐posed.^13^ Second, although diffusion has a direct relation to tissue microstructure, studies have highlighted limitations of microstructure imaging from conventional diffusion encoding (Stejskal‐Tanner)^14^ alone, as it does not retain information of the microscopic heterogeneity.^8^, ^15^, ^16^ For example, studies showed that using the Stejskal‐Tanner experiment (or linear tensor encoding [LTE]) alone cannot disentangle the effects of multiple isotropic diffusivities, orientation dispersion, and microscopic anisotropy from each other.^17^, ^18^ Therefore, it is difficult to distinguish a tissue with randomly oriented fiber‐like structures from isotropic tissue with varying cell densities, which might reflect different tumor types.^18^

Because these first two limitations lead to degeneracy in multi‐compartment estimation, adding information from additional dimensions has become the main strategy to improve the robustness and accuracy of microstructure estimation. For example, the combination of diffusion and relaxometry has been used in non‐imaging nuclear MR experiments to disentangle different compartments.^19^, ^20^ These approaches have been extended to imaging. For example, incorporating T2 into biophysical models of diffusion improved the specificity of multi‐compartment tissue models,^21^ and estimated intra‐ and extra‐axonal tissue properties.^22^ To address the sensitivity limitation of Stejskal‐Tanner encoding, double diffusion encoding (DDE)^23^ and isotropic diffusion encoding^24^, ^25^ were proposed for probing local pore geometry and for rapid DWI.^26^, ^27^ Westin et al. proposed a general framework to describe diffusion encoding for arbitrary gradient waveforms.^28^ In this framework, the b‐value and encoding direction were replaced by the “b‐tensor,” which also describes the shape of the diffusion encoding, extending the b‐tensor shape from Stejskal and Tanner's linear b‐tensor encoding (LTE) to other shapes like planar and spherical b‐tensor encoding (PTE and STE).^16^, ^18^, ^29^, ^30^, ^31^, ^32^, ^33^, ^34^, ^35^ By using multiple b‐tensor shapes, one can differentiate between, for example, heterogeneity of isotropic compartments and microscopic anisotropy.

As more information or encoding dimensions are needed for multi‐parameter quantification, scan time is the third limitation that prevents microstructure imaging from clinical adoption. To overcome this limitation, Hutter et al. developed an acquisition technique called ZEBRA for a joint sampling of T_1_‐T_2_*‐diffusion^36^ with interleaved inversion times and diffusion encodings from multiple scans, and Ma et al. developed an efficient T1, T2, and ADC mapping method using Multi‐tasking.^37^ However, none of these studies have explored the scan efficiency of tensor‐valued diffusion encoding for joint relaxation‐diffusion imaging. Specifically, because the STE gradients are isotropic diffusion‐sensitizing gradients that can directly provide the trace of the diffusion tensors from a single scan, the scan with STE gradients could be potentially more efficient in quantifying ADC as compared to the scan with linear tensor encoding (LTE, or Stejskal‐Tanner) that requires directional averaging. Although studies in the 90s^24^, ^25^ have already identified this benefit and proposed optimization methods for STE, the performance and application were still limited by hardware constraints.

In this study, we developed a multidimensional MR fingerprinting scan (mdMRF) to address the three limitations mentioned above, that is to simultaneously quantify multiple tissue properties that are sensitive to tissue microstructure in a clinically feasible time. MR fingerprinting (MRF) is a fast quantitative imaging technique that is able to quantify multiple tissue property maps simultaneously from a single scan.^38^ Specifically, MRF provides a highly flexible framework to encode MR signals with multiple acquisition parameters, such as variable flip angles, TI, TE, and TR times, diffusion b‐values and encoding directions. The signals that are sensitive to multiple tissue properties, such as relaxation and diffusion, are then varied without reaching a steady state. This encoding strategy has shown improved robustness and accuracy in tissue property quantification^39^, ^40^, ^41^, ^42^ and multi‐compartment separation.^43^, ^44^ Moreover, dictionary‐based MRF mapping has shown high tolerance to measurement noise and artifacts (aliasing and motion), allowing accurate and reproducible tissue property estimation even under an acceleration rate of up to ∼400 as compared to the Nyquist rate.^38^, ^39^, ^40^, ^42^, ^45^, ^46^ Both features will contribute to a clinically feasible solution for multidimensional imaging. Because different b‐tensors require somewhat different sequence designs, such as the minimum TE and number of diffusion encoding directions, for the measurement of ADC (or mean diffusivity), we additionally studied the scan efficiency, scan time, and image quality of using two different b‐tensor encodings in the mdMRF scans, including linear tensor encoding and spherical tensor encoding using optimized gradient waveform designs,^47^, ^48^ for joint relaxation and diffusion quantification. Here, we demonstrate that mdMRF enables simultaneous quantification of T1, T2, and ADC maps in 24 s per slice.

## 2 METHOD

### 2.1 Pulse sequence design

**FIGURE 1:**
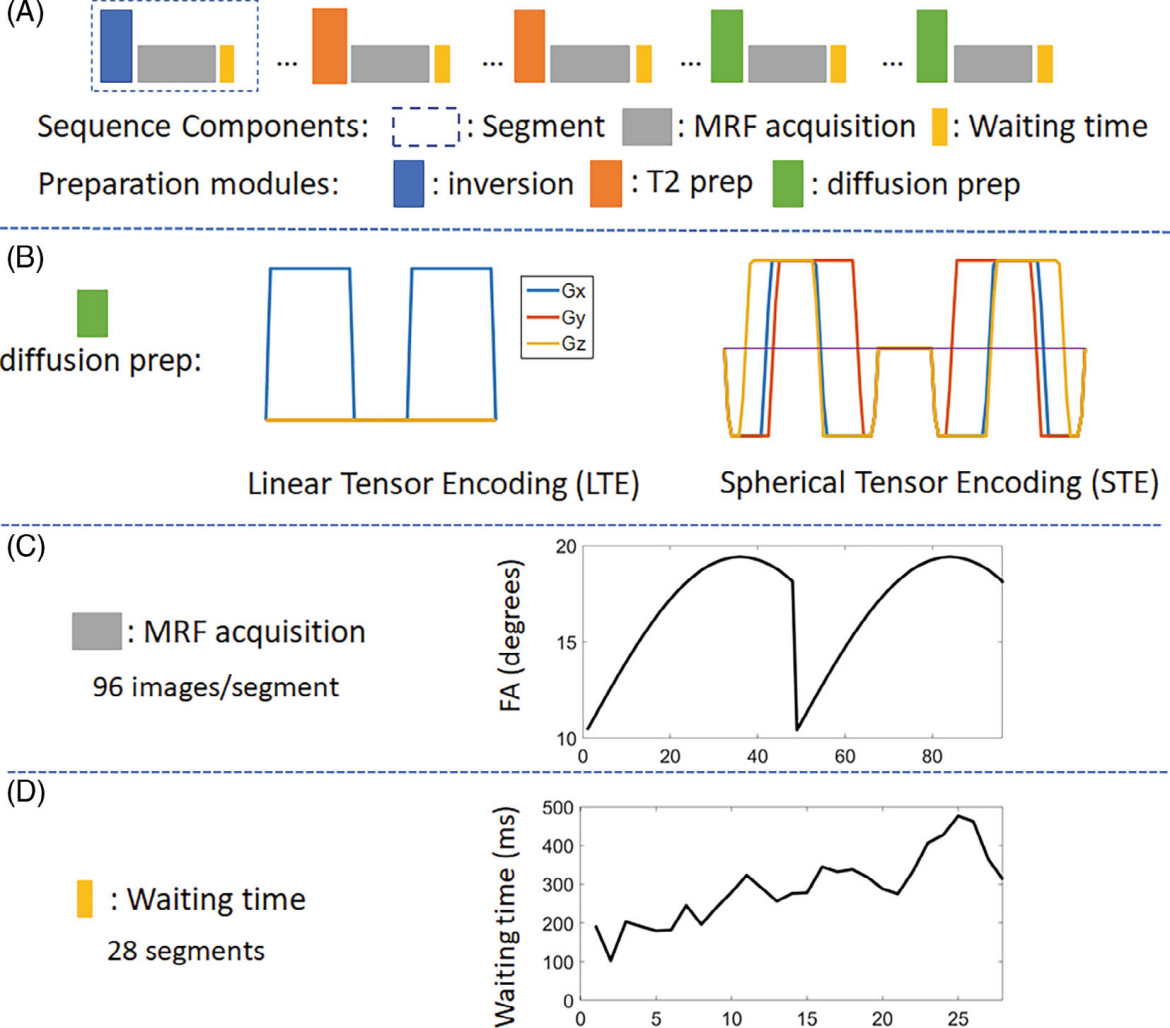
The sequence structure of a multidimensional MRF (mdMRF) scan. A, Acquisition segments, including T1 inversion pulses, T2 preparations, diffusion preparations and MRF readouts. B, The gradient waveforms for linear and spherical tensor encoding. C, An example of a flip angle pattern used in the mdMRF scans. D, An example of the waiting time pattern added at the end of each segment

Figure 1 demonstrates the sequence structure of an mdMRF scan. The sequence is composed of multiple acquisition segments, each starting with a preparation module such as T1 inversion pulses, T2 preparations using MLEV (Malcolm‐Levitt) composite pulses,^49^ and diffusion preparations with adiabatic excitation and refocusing pulses (BIR‐4) and diffusion encoding gradients. Various TIs, TEs, b‐values, and diffusion encoding directions can be implemented in the preparation modules in order to encode the signals with multidimensional parameters. In this study, we implemented mdMRF with linear tensor encoding (mdMRF‐LTE) and spherical tensor encoding (mdMRF‐STE) separately. The diffusion encoding gradient waveforms are shown in Figure 1B. The STE gradient waveform was designed using the NOW toolbox with concomitant field compensation,^47^, ^48^, ^50^ which showed a 22% reduction of the TE time to achieve the same b‐value as compared to the conventional design. After each preparation module, a series of MRF images was acquired, followed by a waiting time to allow the spins to partially recover before the next acquisition segment. One set of preparation module, MRF readouts, and waiting time make up an acquisition segment shown in Figure 1A. In each MRF readout, a single‐shot uniform‐density spiral trajectory was used with an undersampling factor of 48 and a readout duration of 3 ms. The spiral was rotated with a golden angle from one image to the next. Figure 1C shows one example of a flip angle pattern used in each segment where 96 images were acquired. Figure 1D shows an example of the waiting time pattern added at the end of each segment, where 28 segments were acquired from a single mdMRF scan. The waiting time was varied ranging from 100 ms to 500 ms. The variation was added to increase the variation of the segment duration and to avoid acquiring the images at a constant timing in pulsation cycles. A 100 ms lower bound was added to avoid high specific absorption rate (SAR) due to continuous preparations and excitations and a 500 ms higher bound was chosen to balance the scan time and the variations. For the mdMRF‐LTE scans, the acquisition segments with diffusion encoding were repeated multiple times with three diffusion directions ([1 1–1], [1–1 1], [−1 1 1]). Because STE performs isotropic diffusion encoding,^51^ no rotation was applied to the diffusion encoding gradients in the mdMRF‐STE scans. Two example sequence patterns, including flip angles, the order and values of all the preparation modules and timings, are listed in Supporting Information Table S1, which is available online.

Diffusion‐weighted scans can be highly sensitive to phase errors, such as those resulting from eddy current and physiological motions (cardiac pulsation). In this study, three MRF readout designs (gray section of Figure 1A) were implemented to investigate the robustness of the resulting maps to measurement errors: (1) conventional FISP‐based MRF readouts,^52^ (2) FLASH‐based MRF readouts with RF and gradient spoiling, as well as phase stabilizers that are commonly used in the diffusion‐prepared sequences.^37^, ^53^, ^54^, ^55^, ^56^ The phase stabilizers (4pi dephasing) were implemented before the −90 degrees tip‐up pulse of each diffusion preparation module and after each RF pulse of the MRF readout within diffusion acquisition segments. The purpose of the phase stabilizer was to convert the eddy current‐induced phase errors to the signal phase while keeping the signal magnitude unaffected, and (3) FISP and FLASH‐based MRF readouts with peripheral pulsation gating. Specifically, the first MRF readout of each acquisition segment was triggered by the real‐time pulsation signal using a peripheral pulse oximeter placed on the forefinger of the participant's right hand with a 250 ms delay time. The purpose was to avoid the dephasing of signals and phase incoherence between MRF readouts and between acquisition segments due to physiological motion. The waiting time after each segment was set to 0 in all the scans with peripheral pulsation gating, because the pulsation gating introduces subject‐dependent and varying waiting times between segments.

### 2.2 Image acquisition and MRF mapping

All the mdMRF scans, including mdMRF‐LTE and mdMRF‐STE with three different MRF readouts, were implemented on a 3T Prisma scanner (Siemens Healthineers, Erlangen, Germany) and tested in both phantom and healthy volunteers. Five healthy volunteer scans were performed with the approval from the Institutional Review Board. Informed consent was obtained from the volunteers before each scan.

In each scan, 28 acquisition segments, each with 96 MRF images, were acquired. For both phantom and in vivo scans, sequence option 1 in the Supporting Information Table S1, which is available online, was used, which involved interleaved T1, T2, and diffusion preparation modules, varying TI (21 ms), TE (30, 50 ms) and b values (300, 700, 1000 s/mm^2^) with three diffusion directions, and varying flip angles (Figure 1c) and waiting times (Figure 1d) in a single scan. Other acquisition parameters used were: field of view = 300 × 300 mm^2^, in‐plane image resolution = 1.5 × 1.5 mm^2^, slice thickness = 5 mm, TR = 5 ms, acquisition window of each segment = 480 ms, and a series of waiting time ranging between 103 to 476 ms was implemented to make the maximal segment duration to be around 1 s. The scan time was 30 s without peripheral pulse gating and 37.3 ± 3.8 s for scans with peripheral pulse gating.

A dictionary was simulated with a wide range of T1, T2, and ADC, as well as the sequence parameters shown in Supporting Information Table S1 as inputs following the general state equation similar to^57^:

(1) M[n]=A(u[n],θ)M[n−1]+B(u[n],θ)

for n=1,…,N, where *N* is the number of time points (images), M[n] is the signal at the *n*th time point. $A\left(u\left[n\right],\theta \right)\in {\mathbb{R}}^{3\times 3}$ and $B\left(u\left[n\right],\theta \right)\in {\mathbb{R}}^{3\times 1}$ are system matrices determined by RF excitations, relaxation, diffusion, and gradient spoilers where u[n] contains the data acquisition parameters in each time point and θ is the set of tissue properties to be quantified.:

$$\begin{array}{ll} A\left(u\left[n\right],\theta \right) & =G\left(\beta \right)P\left(b,D\right)R\left(T_{1},T_{2},t_{n}\right)Q\left(\alpha_{n},\varphi_{n}\right)\;\text{and}\;\\ B\left(u\left[n\right],\theta \right) & =M_{0}b\left(T_{1},t_{n}\right) \end{array}$$

where $Q\left(\alpha_{n},\varphi_{n}\right)\in {\mathbb{R}}^{3\times 3}$ models the RF excitation with flip angle $\alpha_{n}$ and RF phase $\varphi_{n}$. $R\left(T_{1},T_{2},t_{n}\right)\in {\mathbb{R}}^{3\times 3}$ and $b\left(T_{1},t_{n}\right)\in {\mathbb{R}}^{3\times 1}$ model relaxation during each *TR*, each preparation module, and each waiting period after the acquisition, $P\left(b,D\right)\in {\mathbb{R}}^{3\times 3}$ models diffusion in the diffusion preparation modules and $G\left(\beta \right)\in {\mathbb{R}}^{3\times 3}$ models spin dephasing due to phase stabilizer crushers and gradient spoilers using 250 isochromats. The ranges of the three tissue properties were: T1 from 100 to 3000 ms with a step size of 40 ms, T2 from 10 to 300 ms with a step size of 4 ms, and ADC from 0 to 3 um^2^/ms with a step size of 0.05 μm^2^/ms. The simulation time was 20 min on a standalone PC using Python.

All the mdMRF data were reconstructed using a low‐rank iterative reconstruction^58^ and the resulting images were used to generate T1, T2, ADC and proton density (M0) maps simultaneously using dictionary matching. For the mdMRF scans with LTE, the low‐rank reconstruction and mapping were performed in each of the three directions separately first, which resulted in T1, T2, and diffusion coefficients of three directions D1, D2, and D3. The mean diffusivity was then calculated by averaging the three diffusion coefficients. Because there was no rotation of the diffusion encoding gradients in the scans with STE, the reconstruction was performed directly to all the images that were then matched to the entire dictionary to generate T1, T2, ADC, and M0 maps.

### 2.3 Data analysis

The accuracy of the quantitative results of the mdMRF scans was compared with alternative mapping strategies, including conventional MRF scans, as well as diffusion scans with single‐shot EPI readout, performed at the same slice location. The scan parameters were: MRF: FOV 300 × 300 mm^2^, image resolution 1.2x1.2 mm^2^, slice thickness = 5 mm, acquisition time 20 s. Diffusion: FOV 256 × 256 × 102 mm^3^, image resolution 2 × 2 × 2 mm^3^, TE/TR = 92/7800 ms, partial Fourier factor 5/8, 71 volumes with b = 1000 s/mm^2^ and 8 with b = 0, NEX = 2, TA = 21 min. For the phantom reference scans, we used both NIST relaxation phantom and diffusion phantom because the T2 values of all the tubes in the diffusion phantom are above 500 ms, which is way beyond the physiological range.

The scan efficiency between the mdMRF‐LTE and ‐STE was compared. The scan efficiency was estimated based on the measure of the precision of each tissue property per square root of time^38^:

$$\text{Efficiency}=\frac{\mathrm{QNR}}{\sqrt{T_{\text{scan}}}}$$

where Q is the quantitative tissue properties, such as *T1*, *T2*, and *ADC*, *QNR* is the *T1*, *T2*, and *ADC* to noise ratio, defined by the mean value divided by the estimated error, and *T*_*scan*_ is the scan time. The quantitative estimation of the estimated errors and efficiency were calculated pixel‐wise using a bootstrapped Monte Carlo method.^59^ An additional noise scan was performed using the same sequence but with no RF excitations. Then 50 reconstructions were performed by randomly resampling the acquired noise and adding it to the raw data of the mdMRF scan. The means and SD of T1, T2, and ADC along 50 repetitions were calculated.

To evaluate the phantom experiments, nine regions of interest (ROIs) were drawn on the NIST relaxation phantom and 13 ROIs were drawn on the diffusion phantom. For the in vivo scans, six ROIs were drawn on the frontal and parietal white matter and gray matter regions in both left and right hemisphere of the in vivo results. The results from left and right hemisphere were then averaged, resulting in quantitative estimates of three regions (frontal and parietal white matter and gray matter). Linear regression was performed to evaluate the correlation between the mdMRF results and the reference results. Percentage bias was evaluated for each ROI between the mdMRF scans and the reference scans according to (bias% = Q_MRF_‐Q_ref_)/Q_ref_*100%). Paired t‐tests were performed to evaluate whether there is a significant difference in the results between mdMRF‐LTE and mdMRF‐STE scans.

## 3 RESULTS

**FIGURE 2:**
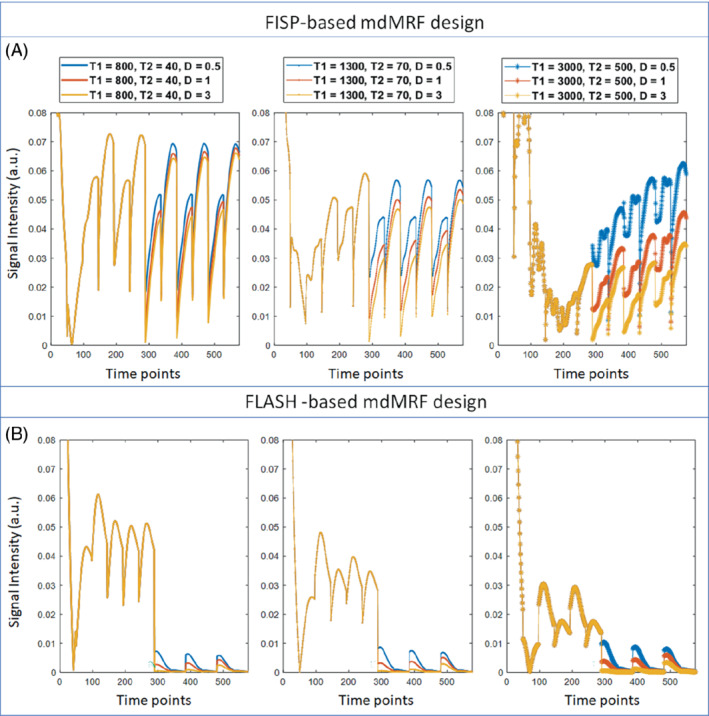
The signal evolutions simulated from the mdMRF scans with the FISP‐based (A) and FLASH‐based (B) MRF readout designs. The first 288 time points were from three acquisition segments with T1 and T2 preparations while the latter 288 time points were from three segments with diffusion preparations with b = 1000, 700, and 300 s/mm^2^, respectively. Signals from each column have the same T1 and T2 values based on white matter (T1 = 800 ms, T2 = 40 ms), gray matter (T1 = 1300 ms, T2 = 70 ms), and CSF (T1 = 3000 ms, T2 = 500 ms), and different colors represent different diffusion coefficient values (D = 0.5, 1, and 3 um^2^/ms)

Figure 2 compares the signal evolutions simulated from the mdMRF scans with the FISP‐based (a) and FLASH‐based (b) MRF readout designs, both using the sequence parameter design (option 1) listed in Supporting Information Table S1. The first 288 time points were from three acquisition segments with T1 and T2 preparations while the latter 288 time points were from three segments with diffusion preparations with b = 1000, 700, and 300 s/mm^2^, respectively. Signals from each column have the same T1 and T2 values based on white matter (T1 = 800 ms, T2 = 40 ms), gray matter (T1 = 1300 ms, T2 = 70 ms), and CSF (T1 = 3000 ms, T2 = 500 ms), and different colors represent different diffusion coefficient values (D = 0.5, 1, and 3 um^2^/ms). The signals from the FISP‐based MRF readout designs (Figure 2A) have much higher signal intensity than those from the FLASH‐based designs (Figure 2B) in the segments with diffusion preparation. In addition, the signals decay away during each MRF acquisition window in the FLASH‐based design, rather than recovering back in the FISP‐based design.

**FIGURE 3:**
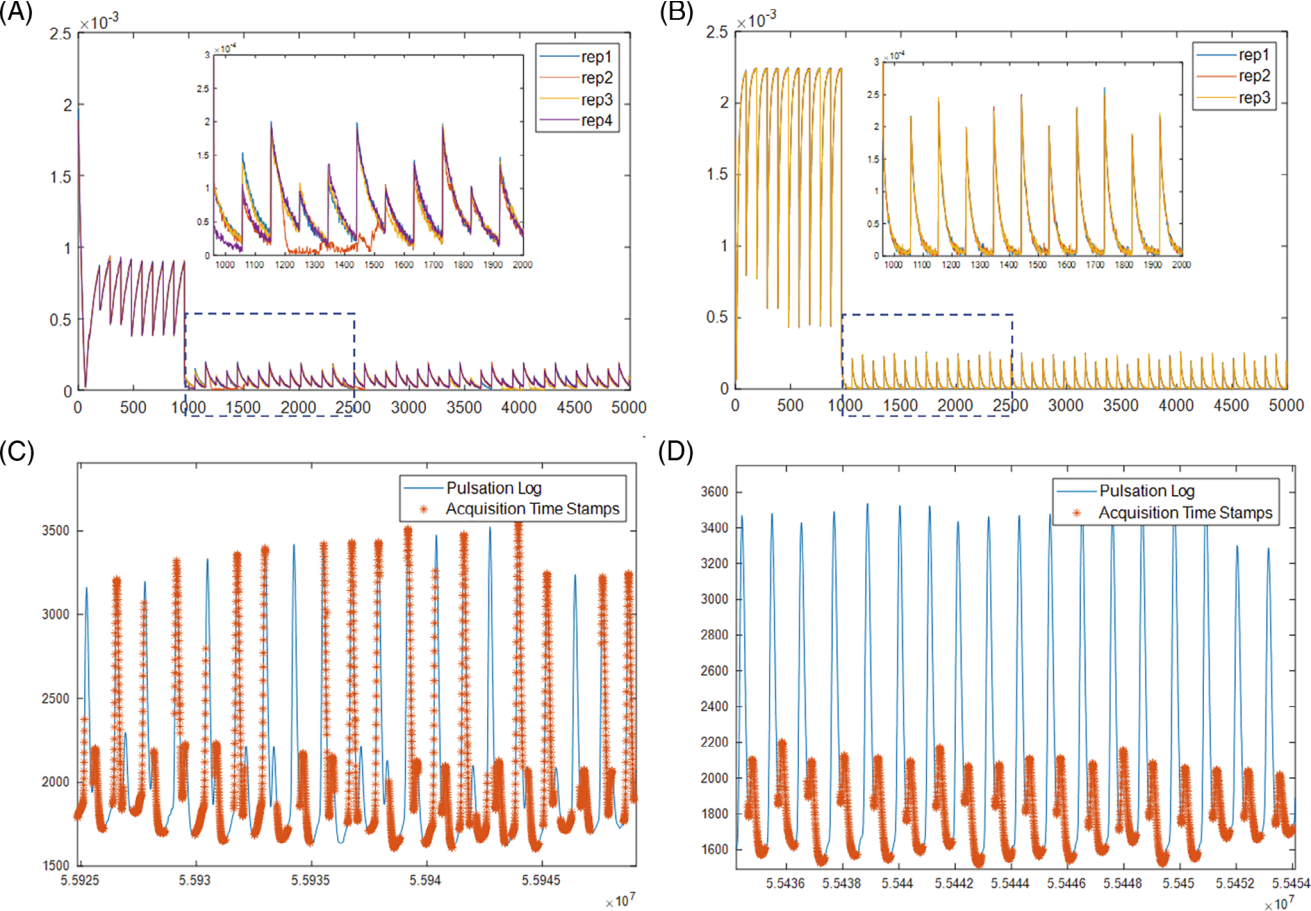
The observation of the signal errors due to physiological motion and the implementation of peripheral pulsation gating. A, The acquired signals (central k‐space) of an in vivo mdMRF scan with four repetitions. B, The signals acquired from a post‐mortem brain using the same mdMRF scan with three repetitions. C, The time stamps of the acquisition windows from an mdMRF scan without pulsation gating, overlaid on the pulsation log of the scans. D, The acquisition with pulsation gating with a 250 ms trigger delay

Figure 3 summarizes the observation of the signal errors due to physiological motion and the implementation of peripheral pulsation gating. Figure 3A shows the acquired signals (central k‐space) of an in vivo FLASH‐based mdMRF scan with constant flip angles of 10 degrees (sequence design option 2 in Supporting Information Table S1) and with four repetitions. Sudden signal drop‐outs are observed at random locations in the acquisition segments with diffusion preparation. As shown in the zoom‐in view, the signal drops can be in the middle of the acquisition window, which indicates that the error is not due to sequence design or preparation modules. As a comparison, Figure 3B shows the signals acquired from a post‐mortem brain using the same mdMRF scan with three repetitions. There is no signal difference among the three repetitions. Therefore, the signal drop‐outs could be due to motion (peripheral pulsation and/or bulk motion) from the volunteer scan. The signal amplitude from the post‐mortem scan is higher than that from the in vivo scan, due to altered relaxation and diffusion coefficients in the post‐mortem brain. Figure 3C and D compare the timestamps of the acquisition windows from an mdMRF scan without and with pulsation gating, overlaid on the pulsation log of the scans. Without pulsation gating, the acquisition windows of the mdMRF scan can start anywhere in the pulsation cycle. On the other hand, Figure 3D shows the acquisition with pulsation gating with a 250 ms trigger delay. The gating ensures that the acquisition windows start at the same phase of the cardiac cycle and avoid the systolic part.

**FIGURE 4:**
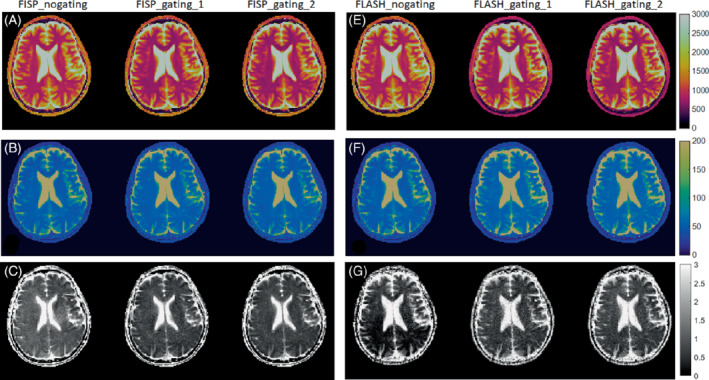
The in vivo T1, T2, and ADC maps acquired from mdMRF‐LTE scans with different sequence designs. A–C, The T1, T2, and ADC maps from FISP‐based MRF readout designs with and without pulsating gating (FISP‐gating‐1, 19 acquisition segments, scan time = 24 s; FISP‐gating‐2, 28 acquisition segments, scan time = 34 s). E–G, The T1, T2, and ADC maps from FLASH‐based MRF designs with and without pulsation gating

Figure 4 compares the in vivo T1, T2, and ADC maps acquired from mdMRF‐LTE scans with different sequence designs. Specifically, Figures 4A–C compare the maps from FISP‐based MRF readout designs with and without pulsating gating. The maps from a shorter scan time (FISP‐gating‐1, 19 acquisition segments, scan time = 24 s) are also compared to those from the longer scan time (FISP‐gating‐2, 28 acquisition segments, scan time = 34 s). Similarly, Figure 4E–G compare the maps from FLASH‐based MRF designs with and without pulsation gating. In general, there are no artifacts observed in T1 and T2 maps from all the scans, except that the T1 values are slightly overestimated and the T2 values are underestimated from the scans with no gating. There are clear “shading artifacts” in the ADC maps from both FISP and FLASH based mdMRF scans without pulsation gating, where imbalanced ADC values are observed between the left and right hemispheres. The ADC maps from the gated scans show improved image quality without any “shading artifacts.” Compared to the FISP based designs, the FLASH‐based results show higher noise enhancement in the ADC maps (FLASH_gating_1 and FLASH_gating_2) by visual inspection. As a result, the FISP‐based mdMRF scan allows shorter scan time (24 s) with comparable image quality.

**FIGURE 5:**
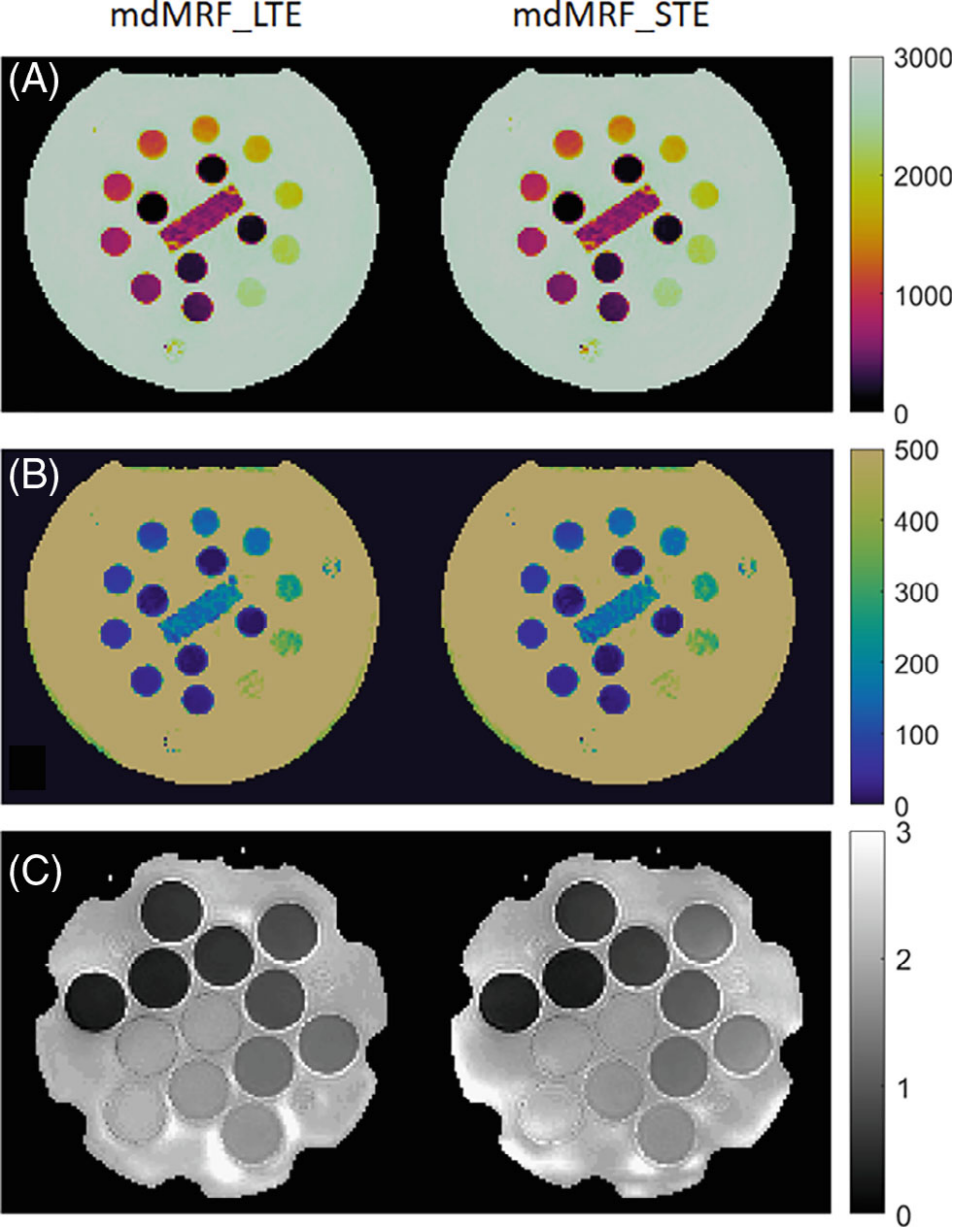
Phantom results. A–C, The T1, T2, and ADC maps acquired from mdMRF‐LTE and mdMRF‐STE scans using FISP‐based sequence design

**FIGURE 6:**
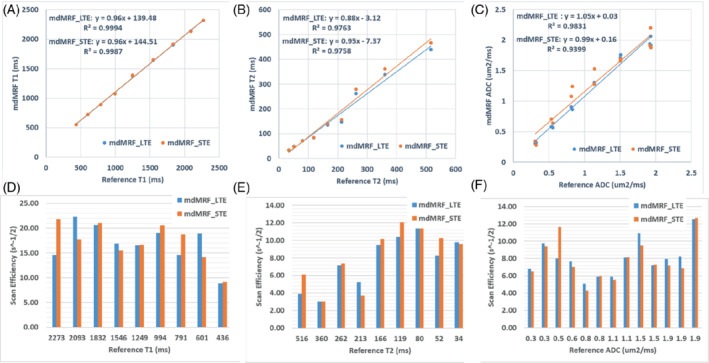
Comparisons of the scan accuracy and efficiency of mdMRF‐LTE and ‐STE scans. A–C, Comparisons of the quantitative T1, T2, and ADC values derived from mdMRF and from the reference scans. D–F Comparisons of the scan efficiency of T1, T2, and ADC from mdMRF‐LTE and ‐STE scans

Figure 5 shows the phantom maps acquired from mdMRF‐LTE and mdMRF‐STE scans using the FISP‐based sequence design. There are high intensity ADC values outside of the diffusion phantom tubes, probably due to B0 shimming or vibration from the distilled water. Figures 6A–C compare the quantitative T1, T2, and ADC values derived from mdMRF and from the reference scans. All the results are in good agreement with the reference values, with all R^2^ above 0.97. T1 from both mdMRF‐LTE and mdMRF‐STE scans show the highest correlation with the reference scans with R^2^ = 0.99. The ADC values from the mdMRF‐STE and ‐LTE scans showed high correlations of R^2^ = 0.93 and R^2^ = 0.98, respectively. There is no significance difference for T1 and ADC between the mdMRF‐LTE and mdMRF‐STE scans (*p* > 0.05 in paired *t*‐test). There is significant difference (*p* = 0.02 in paired *t*‐test) for T2 between mdMRF‐LTE and ‐STE, although the average relative difference among all ROIs is only 3.3%. Between mdMRF and the reference scans, the average biases are 10.4%, −11.0%, and 11.7% for T1, T2, and ADC values, respectively. Figures 6D,E, and F compare the scan efficiency between mdMRF‐LTE and mdMRF‐STE scans. The average scan efficiencies for mdMRF scans are 16.9, 7.7, 8.0 for T1, T2, and ADC from the mdMRF‐LTE scan and are 17.3, 8.3, and 7.8 (s^−1/2^) for T1, T2, and ADC from the mdMRF‐STE scan. There is no significant difference (*p* > 0.05 in paired t‐test) for the scan efficiencies of all tissue properties between the mdMRF‐LTE and ‐STE scans.

**FIGURE 7:**
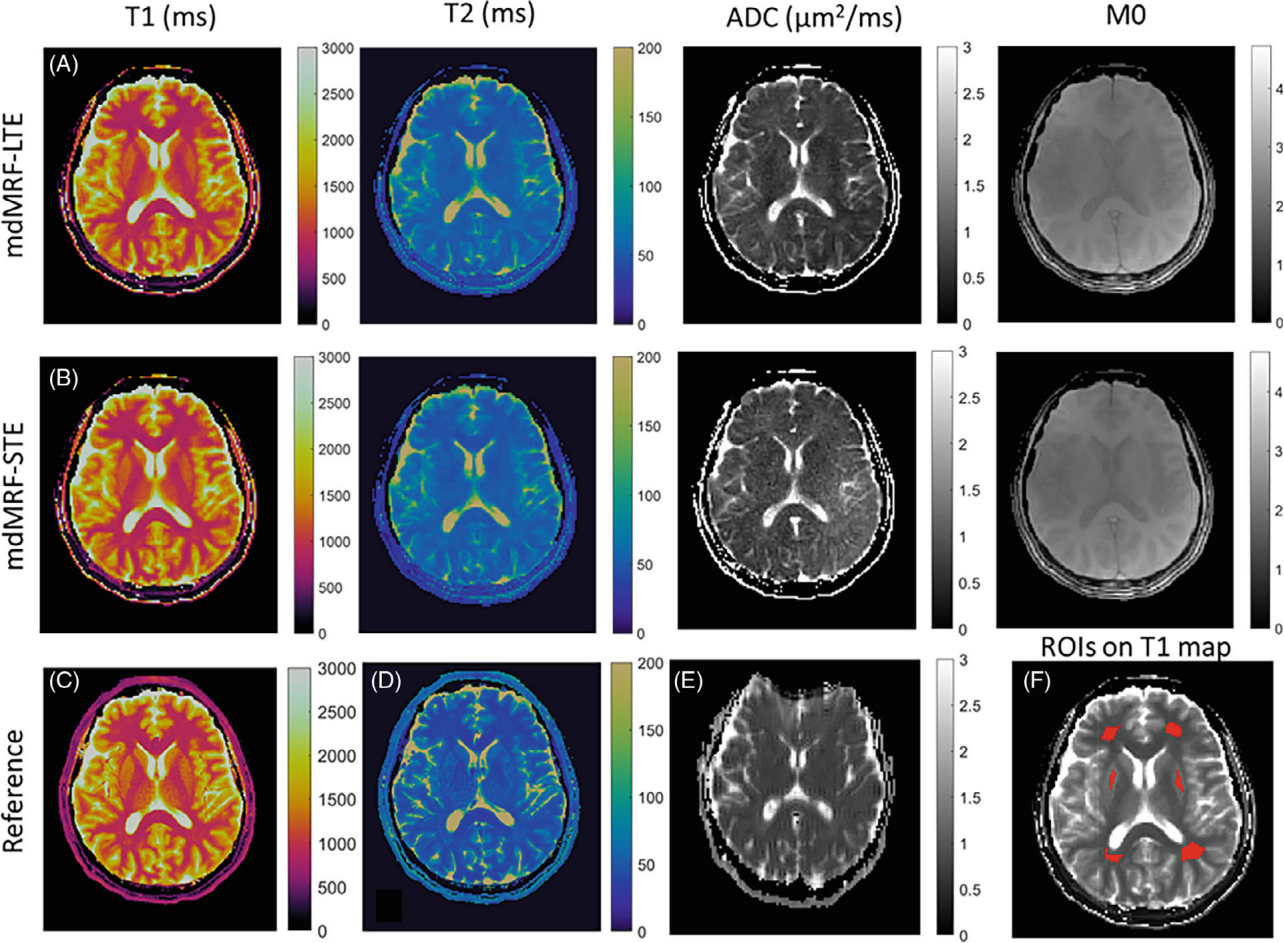
The in vivo T1 (ms), T2 (ms), ADC (μm^2^/ms), and proton density (M0) maps acquired from mdMRF‐LTE (A) and mdMRF‐STE (B) scans with FISP‐based sequence design and pulsation gating. C–E, The reference T1, T2, and ADC maps. F, Six brain ROIs, including four white matter and two gray matter regions overlayed on the T1 map

Figure 7 shows the in vivo maps acquired from mdMRF‐LTE (A) and mdMRF‐STE (B) scans with FISP‐based sequence design and pulsation gating from one of the volunteer scans. The reference T1, T2, and ADC maps are shown in Figures 7C–E. Six brain ROIs shown in Figure 7F were then drawn in the T1, T2, and ADC maps from four healthy volunteers, including ROIs in the frontal, parietal white matter, and gray matter. The quantitative values and the reference values are shown in Table 1. There is no significant difference for the T1, T2, and ADC values between the mdMRF‐LTE and ‐STE scans. Between mdMRF and the reference scans, the average biases are −2.9%, 23.6%, and −12.9% for T1, T2, and ADC from the mdMRF‐LTE scan, respectively, and are −2.3%, 23.1%, and −4.1% for T1, T2, and ADC from the mdMRF‐STE scan, respectively. The scan efficiency of T1, T2, and ADC of one of the volunteer scans were estimated. The average scan efficiencies are 9.5, 6.4, and 2.9 for T1, T2, and ADC from the mdMRF‐LTE scan, and are 10.5, 5.9, and 1.8 (s^‐1/2^) for T1, T2, and ADC from the mdMRF‐STE scan.

**TABLE 1:** Quantitative values of T1, T2, and ADC from mdMRF‐LTE and ‐STE scans from four volunteers

N = 4	WM‐Frontal	WM‐Parietal	GM‐Putamen
T1 ref (ms)	900.54 ± 24.86	937.55 ± 46.6	1241.90 ± 57.74
mdMRF‐LTE	879.87 ± 28.57	917.76 ±33.08	1187.92 ± 35.15
mdMRF‐STE	887.27 ± 25.04	921.42 ± 21.11	1195.14 ± 38.37
T2 ref (ms)	36.67 ± 1.78	41.29 ± 3.30	44.51 ± 3.93
mdMRF‐LTE	47.57 ± 0.95	53.26 ± 2.41	49.92 ± 1.42
mdMRF‐STE	47.64 ± 0.16	53.31 ± 1.43	49.15 ± 0.80
ADC ref (μm^2^/ms)	0.77 ± 0.01	0.83 ± 0.04	0.71 ± 0.01
mdMRF‐LTE	0.75 ± 0.06	0.69 ± 0.07	0.56 ± 0.02
mdMRF‐STE	0.76 ± 0.16	0.79 ± 0.17	0.66 ± 0.09

## 4 DISCUSSION

In this study, we demonstrated the feasibility of simultaneously quantifying relaxation and diffusion from a single MR fingerprinting (mdMRF) scan with tensor‐valued diffusion encodings. Because there is a large number of degrees of freedom in designing MRF scans, various sequence designs were implemented and the performances were compared. Overall, the mdMRF scans (mdMRF‐LTE and mdMRF‐STE) with the FISP‐based MRF readouts and pulsation gating provide the best map quality within a scan time of only 24–37 s per slice. The mdMRF‐LTE scans showed lower noise as compared to the maps from the mdMRF‐STE scans. The T1 and T2 maps and the ADC map are perfectly coregistered because they are all from the same scan. In addition, there is negligible image distortion due to field inhomogeneity presented in the ADC maps of all the mdMRF scans.

The scan accuracy and efficiency were compared between the mdMRF‐LTE and ‐STE scans, and between mdMRF scans and the reference scans. For the phantom scans, there was no significant difference in the scan efficiency between mdMRF‐LTE and ‐STE scans. The scan efficiency for T1 was over two times higher than that for T2 and ADC, demonstrating varied tissue sensitivity from the current mdMRF scan implementation. There was also no significant difference in the quantitative values between mdMRF‐LTE and ‐STE scans, except for a 3.3% relative difference in T2 values in the phantom experiment. Between mdMRF scans and the reference scans, there was an average bias of 10% identified in the phantom scans, and up to 20% bias of T2 in the in vivo scan. Because the conventional MRF scans were used as the reference (due to efficient scan time for in vivo scans), the difference could be due to different signal modeling, as the conventional MRF only considered T1 and T2 in the dictionary simulation, while the mdMRF scan simulated T1, T2, and ADC effects. The slice profile effects could also be different, because the flip angle range was between 10 to 60 degrees in conventional MRF and between 10 to 20 degrees in mdMRF scans. The scans with higher flip angles may suffer more from slice profile imperfections. Slice profile and B1 corrections will be implemented in the future to correct for the flip angle‐dependent variations.^60^ In Figure 7, some differences in the ADC maps derived from LTE and STE can be identified. The difference could be due to eddy current induced errors in the STE scan, as the STE gradients have overall higher gradient amplitude and slew rate as compared to the LTE gradients. An eddy current compensated STE waveform will be implemented in the future to address this potential error source.^61^ The difference could also be due to the underlying sensitivity of these two encoding methods to the microstructure heterogeneity. For sufficiently low b‐values, the mean diffusivity (MD) measured by LTE and STE is equivalent. However, as the b‐value increases, the MD estimation is influenced by higher order terms; the LTE variance is affected by the isotropic and anisotropic kurtosis, whereas STE is affected only by the isotropic kurtosis. Thus, in the presence of a positive anisotropic kurtosis (diffusion anisotropy), the MD estimated by LTE is lower than that estimated by STE.

The diffusion encodings were implemented in the mdMRF based on the diffusion‐prepared SSFP (DP‐SSFP)^62^, ^63^, ^64^ where the diffusion encoded signals are tipped up to the longitudinal direction by a −90° pulse and excited by a SSFP acquisition train. A well‐known issue of DP‐SSFP is that the signals acquired in steady state can be more sensitive to phase errors induced by eddy‐currents or motion than other diffusion weighted (DW) acquisitions where image readout follows immediately after the diffusion encoding gradients. First, the phase errors due to eddy current of the diffusion gradients or motion will be stored in the longitudinal direction after the −90‐degree pulse and affect the signal magnitude shot‐to‐shot after being tipped down by the excitation pulses in the SSFP acquisition. Such magnitude inconsistency has been reported to cause signal voids and shading artifacts in the resulting images and ADC maps.^56^, ^65^ Second, if the signal is not fully spoiled in the SSFP acquisition, the phase error may be accumulated in higher order echoes along the long echo train, making it hard to model, identify and correct. Although MRF allows implementation of variable excitations and timings in order to make the signals more incoherent than those from conventional DP‐SSFP, the effects of measurement errors due to eddy current and motions still need to be investigated.

Various approaches have been proposed to address the phase errors for diffusion prepared and multi‐shot diffusion weighted acquisitions. First, stabilizer (or crusher) gradients were introduced to the diffusion prepared sequences to spoil the phase before the tip up pulse in the diffusion preparation and restore it after each excitation in the SSFP acquisition, in order to maintain the correct signal magnitude.^37^, ^54^, ^66^ One can then directly use the magnitude‐only analysis or correct the phase errors separately. The main drawback of this approach is the signal loss due to repeated crusher gradients in each acquisition window, resulting in low SNR of the entire diffusion weighted signal.^37^ Second, artifacts due to pulsatile motion in the diffusion images and the corresponding estimation bias in DTI have been reported.^67^, ^68^, ^69^ The pulsation induced artifacts are spatially varying and are more severe in inferior and medial areas of the brain due to higher velocities.^69^ Although cardiac or pulsation gating reduced such artifacts, such methods suffered from prolonged scan time as compared to non‐gated scans. Third, phase corrections using either navigator‐based^70^, ^71^, ^72^, ^73^ or navigator‐free^74^, ^75^, ^76^ approaches have been commonly used in multi‐shot diffusion scans. In the navigator‐based approaches, the phase errors were estimated using either low‐resolution images acquired separately or from the same scan and corrected in the reconstruction. The navigator free approaches use parallel imaging reconstruction or sparsity constraints to estimate temporal varying phase of each shot and build the phase component into the reconstruction to correct for it.

In this study, we implemented multiple variants of mdMRF and investigated the robustness of each approach. First, there are diffusion specific errors in the resulting maps as shown in Figure 4. The image quality of T1 and T2 maps are consistently good no matter which readout designs or scan time was used, indicating good robustness of mdMRF scans for T1 and T2 quantification. On the other hand, the ADC maps are sensitive to different readout designs. In particular, the ADC maps derived from the non‐gated FISP‐based and FLASH‐based mdMRF scans exhibited image shading (or intensity imbalance), which has been reported previously and has been associated with diffusion sensitivity to phase errors.^53^ Second, as shown in Figure 2, the phase stabilizer approach (FLASH‐based mdMRF) suffered from low signal intensity as compared to FISP‐based MRF design. When comparing the in vivo maps in Figure 4, the ADC maps derived from the FLASH‐based mdMRF scans also showed high noise enhancement than those from the FISP‐based mdMRF scans by visual inspection. The direct consequence is that the FLASH‐based mdMRF scans require longer scan time to achieve good map quality. Third, the phase stabilizer approach was not able to fully address the measurement errors, shown in Figure 4g, while adding pulsation gating was able to restore the image quality from both FISP‐based and FLASH‐based mdMRF scans.

The main concerns of the pulsation gated scans are the scan efficiency and the extension to volumetric acquisitions. Nevertheless, the waiting time was added in between acquisition segments to allow the spins to recover, so there is no significant increase of scan time between the non‐gated and gated scans. The waiting times between segments were varied, based on the empirical design in non‐gated scans and pulsation triggering in gated scans. The dictionary simulation was able to flexibly account for spin relaxation during varying waiting times. As shown in the results of in vivo scans, no significant difference was identified in T1, T2, and ADC maps between scans, even though each scan had different timing due to subject‐dependent pulsation gating. The waiting time of the non‐gated scans could be improved, so the phase errors due to physiological motion are incoherent across hundreds of MRF images. When mapping the tissue properties using a dictionary with ideal signal evolutions, the more incoherent phase errors may lead to a less biased estimation, similar to the high robustness of MRF to aliasing artifacts and spiral gradients induced errors shown in previous works.^38^ Design and optimization of the diffusion gradients^50^, ^51^, ^77^ in terms of, for example, eddy‐current reduction can be implemented to further improve image quality and parameter accuracy.^61^, ^77^ The mdMRF pulse sequence can also be further optimized because the current implementations, including excitations, timing, location, and values of the preparation modules, were heuristically designed. Our group proposed a physics‐inspired optimization framework for MRF recently that uses a cost‐function based on explicit first‐principle simulation of systematic errors arising from undersampling and phase errors.^78^ This framework will be applied to optimize mdMRF for better quantification of relaxation and diffusion simultaneously. In terms of extending the current implementation to volumetric acquisitions, fast imaging techniques, such as simultaneous multi‐slice acquisition,^79^, ^80^, ^81^ 3D sampling strategies,^39^, ^40^ and optimized interleaved scans^36^, ^69^ will be investigated. Finally, phase correction techniques proposed previously are promising approaches to address FISP‐based acquisition without pulsation gating. Because variable density spiral trajectories can be implemented in the mdMRF scans, the phase errors may be estimated first by reconstructing low resolution images from densely sampled spirals and corrected using low‐rank or sparsity constraint reconstructions.^74^

Finally, in this study, we implemented mdMRF scans with tensor‐valued diffusion encoding, including linear and spherical b‐tensors. Previous work showed that tensor‐valued diffusion encoding may bring additional information to investigations of schizophrenia,^28^ brain tumors,^18^, ^82^, ^83^ multiple sclerosis,^84^ cortical malformations,^85^ prostate tumors,^86^ healthy brain,^8^, ^87^ and kidneys.^88^ In these studies, the signal from LTE and STE scans were used to, for example, extract the microscopic diffusion anisotropy which is sensitive to the changes in the microscopic level while it is not influenced by the orientational order of the tissue. Because the main focus of this study was to investigate the feasibility and scan efficiency of using different b‐tensors for the joint relaxation and diffusion quantification, we applied only three diffusion directions in the mdMRF‐LTE scans for the purpose of the ADC measurement. Future work will be to increase the diffusion directions of the LTE and to incorporate both LTE and STE in a single mdMRF scan to provide microscopic anisotropy maps. Both sequence design and multi‐compartment model will be further optimized to efficiently encode and quantify additional tissue dimensions. We acknowledge that the current implementation of mdMRF scans are 2D scans with asymmetric voxels. Asymmetric voxels are known to introduce confounding effects in measures of voxel‐scale diffusion anisotropy, for example in DTI, due to the interaction between voxel and structure geometry.^89^ This is especially relevant if the data are also intended to support tractography, where isotropic spatial resolution is preferable.^90^, ^91^ The volumetric implementations especially with 3D volumetric excitation will allow mdMRF scans to have isotropic image resolution and address this concern while providing microscopic anisotropy information.

## 5 CONCLUSIONS

In this study, we developed a multidimensional MRF scan for simultaneous quantification of T1, T2, ADC, and M0 maps in only 24 s per slice. All maps were inherently coregistered without image distortion. Notably, we investigated the feasibility and efficiency of using tensor‐valued diffusion encoding for the ADC quantification. We demonstrated that values measured in both phantom and in vivo were in good agreement with reference measurements. Among various sequence designs, the FISP‐based mdMRF with peripheral pulsation gating provided the best image quality.

## CONFLICT OF INTEREST

Dan Ma and Mark Griswold have MRF patents licensed by Siemens.

## Supporting information


**Appendix S1** Supplementray Information
